# Triglyceride affects the association between estimated glomerular filtration rate and the onset of non-alcoholic fatty liver disease: A second analysis of a Chinese cohort study

**DOI:** 10.3389/fmed.2022.984241

**Published:** 2022-09-27

**Authors:** Haofei Hu, Changchun Cao, Yong Han, Yongcheng He

**Affiliations:** ^1^Department of Nephrology, Shenzhen Second People’s Hospital, Shenzhen, Guangdong, China; ^2^Department of Nephrology, The First Affiliated Hospital of Shenzhen University, Shenzhen, Guangdong, China; ^3^Shenzhen University Health Science Center, Shenzhen, Guangdong, China; ^4^Department of Rehabilitation, Shenzhen Dapeng New District Nan’ao People’s Hospital, Shenzhen, Guangdong, China; ^5^Department of Emergency, Shenzhen Second People’s Hospital, Shenzhen, Guangdong, China; ^6^Department of Emergency, The First Affiliated Hospital of Shenzhen University, Shenzhen, Guangdong, China; ^7^Department of Nephrology, Shenzhen Hengsheng Hospital, Shenzhen, Guangdong, China; ^8^Department of Nephrology, Affiliated Hospital of North Sichuan Medical College, Nanchong, Sichuan, China

**Keywords:** non-alcoholic fatty liver disease, modify, estimated glomerular filtration rate, interactive effect, Cox proportional-hazards regression, triglyceride

## Abstract

**Objective:**

The role of triglyceride (TG) and estimated glomerular filtration rate (eGFR) effect modifiers on the risk of non-alcoholic fatty liver disease (NAFLD) is unknown. This study examined whether TG modifies the relationship between eGFR and incident NAFLD.

**Methods:**

In a Chinese hospital from January 2010 to December 2014, 15,555 non-obese subjects were collected systematically for this retrospective cohort study. The target-independent and dependent variables were eGFR measured at baseline and NAFLD appearing during follow-up. The modified variable was TG measured at baseline. The multivariate Cox proportional hazards model was used to explore eGFR and TG’s association with NAFLD risk. We explored *a priori* interaction between eGFR and TG, and performed subgroup analyses to further assess whether the relationship between eGFR and incident NAFLD was modified by TG. We also explored the effect of TG and eGFR interaction on the risk of NAFLD.

**Results:**

The mean age was 43.09 ± 14.92 years, and 8,131 (52.27%) were males. During a median follow-up time of 35.8 months, 2,077 (13.35%) individuals developed NAFLD. In the adjusted model, eGFR was negatively associated with incident NAFLD (HR = 0.984, 95% CI: 0.982, 0.987), while TG was positively related to NAFLD (HR = 1.582, 95% CI: 1.490, 1.681). TG could modify the relationship between eGFR and incident NAFLD. A stronger association between eGFR and NAFLD could be found in the participants without hypertriglyceridemia (HTG) (HR = 0.981, 95% CI: 0.978–0.984, *P* for interaction = 0.0139). In contrast, the weaker association was probed in the population with HTG (HR = 0.986, 95% CI: 0.983–0.989). At the same time, we also found an interaction between eGFR and TG in influencing NAFLD risk. In participants with decreased eGFR and HTG, the risk of NAFLD was significantly increased. Further, compared to non-HTG subjects with eGFR ≥ 116.56 ml/min/1.73 m^2^, participants with HTG and eGFR < 82.88 ml/min/1.73 m^2^ had about a fourfold increase in the risk (HR = 4.852 95% CI: 3.943–5.970) of NAFLD.

**Conclusion:**

eGFR and TG is independently associated with NAFLD risk. The association of eGFR with incident NAFLD is likely to be modified by TG in the Chinese non-obese population. There was an interactive effect between eGFR and TG in affecting NAFLD risk. In participants with decreased eGFR and hypertriglyceridemia, the risk of NAFLD is significantly increased.

## Background

Non-alcoholic fatty liver disease (NAFLD) is a series of different liver injury syndromes ranging from simple steatosis to non-alcoholic steatohepatitis (NASH). In this case, cirrhosis, liver failure, and hepatocellular carcinoma could eventually develop (1). An estimated one in four adults worldwide suffers from NAFLD, which has become a global health problem that poses a danger to human health (2, 3). Statistically, the prevalence of NAFLD in the general population of China has ranged between 24.77 and 43.91% in recent years (4, 5). And the incidence and prevalence of NAFLD are rapidly increasing (6, 7).

Obesity and NAFLD are closely linked clinically (8, 9). In general, however, people with normal body mass indexes (BMIs) are still diagnosed with NAFLD. The third National Health and Nutrition Examination Survey of America found that 7.4% of non-obese adults had hepatic steatosis (10). A high percentage of this can be found in Asia (8–19%) (11). Additionally, more studies demonstrate that non-obese patients with NAFLD are more likely to suffer from metabolic syndrome and rapidly develop severe liver disease (12, 13). Further, early recognition of non-obese NAFLD could reduce the risk of cardiovascular disease and diabetes (14, 15). Consequently, identifying non-obese people at risk of NAFLD is still important. NAFLD and dyslipidemia are comorbid conditions (16). Studies have shown that low-density lipoprotein cholesterol (LDL-c) has been linked to NAFLD (17, 18). Currently, the prevalence and incidence of NAFLD might be affected by elevated LDL-c levels within the normal range, according to a recent study (19). Due to its increasing prevalence and complexity, we must continue to find new risk factors for the prevention and treatment of NAFLD in China.

As a better and more reliable indicator of kidney filtration function, the estimated glomerular filtration rate (eGFR) is widely used to diagnose chronic kidney disease and evaluate renal function (20). There are many common risk factors associated with NAFLD and CKD, including obesity, dyslipidemia, diabetes, and hypertension (21). The incidence of NAFLD in CKD patients was 4.4%, according to a retrospective cohort study conducted in the United States (22). Another recent research, which included 2,600 Chinese patients with NAFLD and diabetes, found that there was a greater likelihood of liver fibrosis with a lower eGFR (23). Hypertriglyceridemia (HTG) has been identified as a known risk factor for new-onset NAFLD (24). NAFLD is characterized by triglyceride (TG) accumulation in hepatocytes without alcohol abuse (25). Patients with CKD are often accompanied by elevated levels of TG (26). Nevertheless, it is still unclear whether eGFR and TG independently affect the risk of NAFLD or modulate each other to play roles in the risk of NAFLD among adult Chinese non-obese persons. We also do not know whether eGFR and TG play an interactive effect on the risk of NAFLD.

In this study, we will not only examine whether TG modifies the relationship between eGFR and NAFLD risk but also explore whether eGFR and TG play an interactive effect on the risk of NAFLD.

## Materials and methods

### Study design

Data were used from a computerized database created by the Wenzhou Medical Center of Wenzhou People’s Hospital in China for this retrospective cohort study. eGFR was the target-independent variable, while NAFLD was the outcome variable (dichotomous variable: 0 = non-NAFLD, 1 = NAFLD). The modified variable was TG measured at baseline.

### Data source

Raw data were obtained from the DATADRYAD database^1^ provided by Sun et al. data from: Association of low-density lipoprotein cholesterol within the normal range and NAFLD in the non-obese Chinese population: a cross-sectional and longitudinal study, Dryad, Dataset, https://doi.org/10.5061/dryad.1n6c4 (27). Researchers may use Dryad data for secondary analysis without violating authors’ rights under Dryad’s terms of service.

#### Study population

To minimize selection bias, participants were taken from Wenzhou Medical Center in Wenzhou People’s Hospital non-selectively and successively. The participants’ identities were encoded as non-traceable codes to ensure privacy. The hospital’s electronic medical record system was used to retrieve the data. Participants have given written consent to participate in the original study, which was approved by the ethics committee at Wenzhou People’s Hospital (28).

**FIGURE 1:**
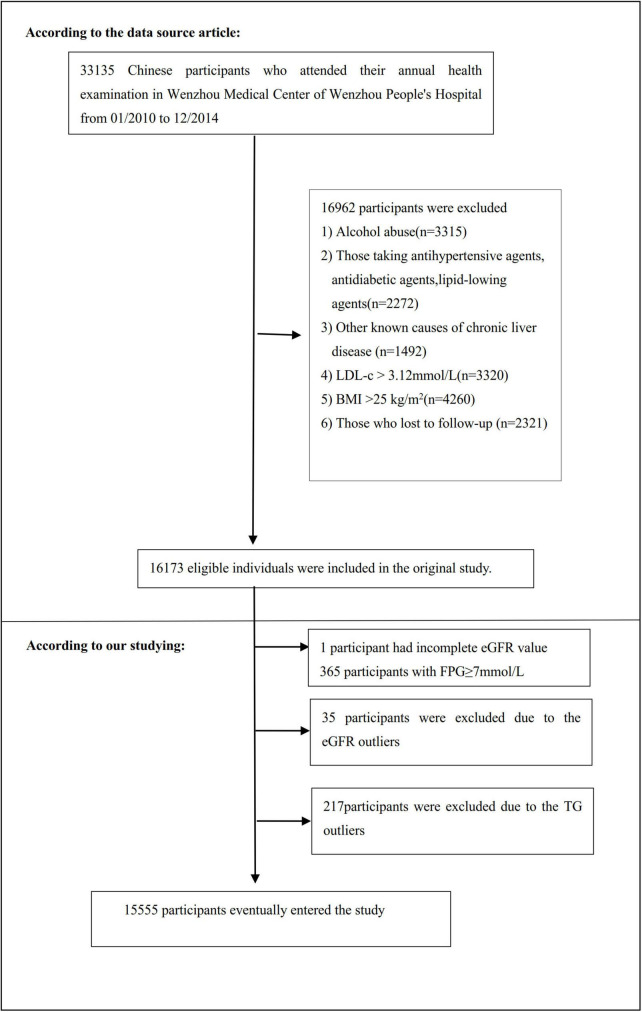
Flowchart of study participants. A total of 16,173 participants were included in the original study. We excluded patients with missing values of eGFR (*n* = 1), FPG ≥ 7 mmol/L (*n* = 365), and outliers of eGFR (*n* = 35) and TG (*n* = 217). The final analysis included 15,555 subjects in the present study.

The study initially included 33,135; thereafter, 17,580 participants were excluded. As a result, 15,555 participants were left to be analyzed (see Figure 1 for details). The Strobe statement was followed for all clinical procedures in this study (29). Inclusion criteria included: Chinese adults who were free of NAFLD and underwent a health examination as part of the longitudinal studies from January 2010 to December 2014 who were free of NAFLD. Exclusion criteria included (28): (1) those consuming excessive amounts of alcohol (above140 g for males and 70 g for females per week); (2) anyone with a history of chronic liver diseases, such as autoimmune hepatitis, NAFLD, or viral hepatitis; (3) those with LDL-c > 3.12 mmol/L and body mass index (BMI) ≥25 kg/m^2^; (4) those taking lipid-lowering, hypertensive, or anti-diabetic medication; and (5) participants who failed to follow up with or who had missing data on BMI, total cholesterol (TC), LDL-c, TG, HDL-c (high-density lipoprotein cholesterol), etc.; (6) participants with FPG ≥ 7 mmol/L and incomplete eGFR; (7) participants with eGFR and TG outliers (outside of the range of means plus or minus three standard deviations) (30, 31).

### Variables

#### Estimated glomerular filtration rate

As a continuous variable, the eGFR was obtained at baseline. According to the Chronic Kidney Disease Epidemiology Collaboration (CKD-EPI) equation for “Asian origin,” eGFR was calculated (32). With the following formula, gender, age, and serum creatinine (Scr) were taken into account:

Scr > 0.7 mg/dL in females, eGFR = 151 × (Scr/0.7)^–1.210^ × 0.993*^age^*;
Scr ≤ 0.7 mg/dL in females, eGFR = 151 × (Scr/0.7) ^–0.328^ × 0.993*^age^*;
Scr > 0.9 mg/dLin Males, eGFR = 149 × (Scr/0.9) ^–1.210^ × 0.993*^age^*;
Scr ≤ 0.9 mg/dL in Males, eGFR = 149 × (Scr/0.9) ^–0.415^ × 0.993*^age^*;

Age and Scr are measured in years and mg/dL, respectively. With this new Asian CKD-EPI equation, more accurate GFR estimates for Chinese patients with CKD may be possible, particularly in populations with higher GFRs. CKD was defined as eGFR < 60 mL/min/1.73 m^2^ for 90 days (22).

#### Triglyceride

Standard methods were used to measure TG using an automated analyzer (Abbott AxSYM). Hypertriglyceridemia (HTG) is defined as serum levels of TG ≥ 1.7 mmol/L (33). We divided participants into hyperglycemic and non-hyperglycemic groups based on triglyceride levels.

#### Outcome measures

According to the Chinese Liver Disease Association, participants were diagnosed with NAFLD using ultrasonography (34). Particularly, NAFLD was diagnosed based on five criteria: (1) The near-field echogenicity in the liver region was diffusely increased, and the far-field echogenicity was gradually decreased; (2) Intrahepatic cavity structure was unclear; (3) Mild to moderate hepatomegaly with rounded margins; (4) Decreased hepatic blood flow signal; (5) The right lobe of the liver and the diaphragmatic capsule was poorly or incompletely developed (28).

During the observation period, annual follow-up assessments were conducted. NAFLD risk was determined by performing liver ultrasonography in a blinded manner (as at baseline). Participants were censored at either the last visit or the time of diagnosis of NAFLD. Follow-up lasted for five years.

#### Other variables

There were also the following other variables in the database: (1) continuous variables: BMI, age, alanine aminotransferase (ALT), TC, diastolic blood pressure (DBP), direct bilirubin (DBIL), albumin (ALB), HDL-c, γ-glutamyl transpeptidase (GGT), systolic blood pressure (SBP), globulin (GLB), aspartate aminotransferase (AST), total bilirubin (TB), LDL-c, blood urea nitrogen (BUN), alkaline phosphatase (ALP), and fasting plasma glucose (FPG); (2) categorical variables: gender.

The biochemical values were analyzed with an automated analyzer (Abbott AxSyM) according to standard methods. Health habits and medical history were assessed by a physician (28). A person’s BMI was calculated by dividing their metric height by their metric weight (kg/m^2^). Based on the Chinese criteria for obesity, the BMI was categorized according to predefined standard categories (35): underweight (<18.5 kg/m^2^), normal weight (18.5 to <24.0 kg/m^2^), overweight (24.0 to <28.0 kg/m^2^). FPG of 6.1–6.9 mmol/L was considered to be impaired fasting glucose (IFG), according to the World Health Organization (WHO) (36). When FPG exceeded 7 mmol/L, diabetes was considered (37). The presence of ALT > 40 U/L indicated liver dysfunction (38). The grouping variable was age, split at 10 years (39–41). The previous reports provided more specific details (28, 42).

#### Statistical analysis

Participants were stratified by eGFR quartile into Q1 (<82.88 ml/min/1.73 m^2^), Q2 (≥82.88, <99.70), Q3 (≥99.70, <116.56), and Q4 (≥116.56) groups (43–45). For continuous variables, the mean (standard deviation) or median (range) (non-normal distribution) was used, and for categorical variables, the number (%) was used. Testing for differences among eGFR groups was done using the one-way ANOVA method (normal distribution), the χ^2^ method (categorical variables), or the Kruskal–Wallis H test (skewed distribution). The Kaplan–Meier method was used to compute survival estimates and time-to-NAFLD variables. Log-rank tests were conducted to compare the Kaplan–Meier probability of survival free of NAFLD among eGFR groups.

In order to better explore the relationship between eGFR, TG and NAFLD risk, we mainly conducted the following three-step analysis. First, to explore the association between eGFR, TG and NAFLD, respectively. Based on our analysis of collinearity, we created three models using univariate and multivariate Cox proportional-hazards regression models, including a non-adjusted model (Crude model: no covariates were adjusted), a minimally-adjusted model (Model I: only sociodemographic variables, such as gender, age, SBP, BMI, and DBP were adjusted) and a fully adjusted model (Model II, including DBP, FBG, GLB, SBP, HDL-c, BMI, ALB, age, LDL-c, BUN, sex, and ALT). The effect sizes (HR) were calculated with 95% confidence intervals (CI). Our model adjusted them when covariances were added, and the hazard ratio (HR) changed by 10% or more (29). The screening for collinearity was also considered. We excluded TC from the multivariate Cox proportional-hazards regression equation based on the results of the collinearity screening because it was collinear with other variables. We also estimated the associations between TG and eGFR in the entire cohort using the univariate and multivariate linear regression model with the same adjustment strategy.

Second, the modification effect of TG on the relationship between eGFR and the risk of NAFLD was explored. We performed a stratified univariate and multivariate Cox proportional hazards regression model based on whether participants had hypertriglyceridemia. Testing for interactions was done using the likelihood ratio test for models that included and did not include interaction terms (46, 47). If the interaction test was statistically significant, it suggested that TG could modify the relationship between eGFR and NAFLD. Since the risk of NAFLD was obviously increased in patients with CKD (48). As a result, when examining the effect of TG on the relationship between eGFR and NAFLD, participants with eGFR < 60 mL/min/1.73 m^2^ were excluded from sensitivity analyses.

Third, to explore whether eGFR and TG play an interactive effect on the risk of NAFLD. We divided all participants into eight groups based on eGFR quartiles and hypertriglyceridemia. Using non-hypertriglyceridemia participants with eGFR ≥ 116.56 ml/min/1.73 m^2^ (Q4) as a reference, we analyzed the effects of the other seven groups on the risk of NAFLD by univariate and multivariate Cox proportional hazards regression models. We determined the interaction of TG and eGFR on the NAFLD risk by comparing effect sizes between different groups.

An analysis of the subgroups was also conducted using a stratified Cox proportional-hazards regression model across a wide range of variables (FPG, gender, BMI, DBP age, SBP, and ALT) among participants with HTG or not. Firstly, continuous variables, such as BMI (<18.5, ≥18.5 to <24, ≥24 kg/m^2^), SBP (<140, ≥140 mmHg), age (<30, ≥30 to <40, ≥40 to <50, ≥50 to <60, ≥60 to <70, ≥70 years), ALT (≤40, >40 U/L), FPG (≤6.1, >6.1 mmol/L), DBP (<90, ≥90 mmHg) (49) were converted to categorical variables on the basis of the clinical cut point. Secondly, each stratification was adjusted for all factors in addition to the stratification factor itself (DBP, FBG, GLB, SBP, HDL-c, BMI, ALB, age, LDL-c, BUN, sex, and ALT). As the last step, interaction tests with and without interaction terms were conducted using the likelihood ratio test (46, 47).

The number of participants with missing data of ALP, GGT, ALT, AST, ALB, GLB, TB, DBIL, SBP, and DBP were 4,001 (25.7%), 4,003 (25.7%), 4,001 (25.7%), 4,001 (25.7%), 1,335 (8.6%), 1,335 (8.6%), 5,526 (35.5%), 6,890 (44.3%), 19 (0.1%), and 19 (0.1%), respectively. Missing covariant data were handled via multiple imputations (50). The imputation model included AST, age, ALB, DBIL, SBP, ALP, LDL-c, DBP, TC, GLB, sex, HDL-c, ALT, TG, BMI, BUN, FBG, TB, and GGT. The missing data analysis procedure was based on the missing-at-random assumption (MAR) (51). Based on the STROBE statement, all results were written (29).

Analysis was conducted using R^2^ (The R Foundation) and EmpowerStats^3^ (X&Y Solutions, Inc, Boston, MA). Statistical significance was defined as a *P* value less than 0.05 (two-sided).

## Results

### Baseline characteristics of participants

TG and eGFR stratified baseline clinical and biochemical characteristics of participants were presented in Table 1. The mean age was 43.09 ± 14.92 years old, and 8,131 (52.27%) were males. The mean baseline eGFR and TG were 99.17 ± 22.76 ml/min per 1.73 m^2^ and 1.23 ± 0.61 mmol/L, respectively. During a median follow-up time of 35.83 months, 2,077 (13.35%) people experienced NAFLD. Based on the triglyceride levels, we first divided all participants into HTG (TG ≥ 1.7 mmol/L) and non-HTG (TG < 1.7 mmol/L) groups. Participants in each group were then subdivided into four subgroups based on eGFR quartiles (<82.88, ≥82.88 to <99.70, ≥99.70 to <116.56, ≥116.56). In the TG < 1.7 mmol/L group, when compared with the Q1 (<82.88) subgroup, the levels or proportions of males, GLB, and HDL-c increased significantly in the Q4 (≥116.56) group. In contrast, the opposite results were found in covariates in terms of females, age, GGT, BMI, SBP, AST, TG, DBP, FPG, TC, LDL-c, ALP, Scr, BUN, ALT, TB, and DBIL. While in the TG ≥ 1.7 mmol/L group, compared with the Q1 (<82.88) subgroup, individuals had lower levels or proportions of female, age, BMI, SBP, GGT, AST, DBP, ALT, TG, FPG, Scr, ALP, TB, BUN, and higher levels of HDL-c in the Q4 subgroup.

**TABLE 1:** The baseline characteristics of participants.

	TG < 1.7 mmol/L					TG ≥ 1.7 mmol/L				
			
eGFR Quartile	Q1 (<82.88)	Q2 (82.88–99.70)	Q3 (99.70–116.56)	Q4 (≥116.56)	*P*	Q1 (<82.88)	Q2 (82.88–99.70)	Q3 (99.70–116.56)	Q4 (≥116.56)	*P*
*N*	2910	3116	3302	3496		978	769	587	397	
Age (years)	48.9 ± 16.0	47.2 ± 16.6	42.9 ± 12.5	33.8 ± 8.1	<0.001	49.3 ± 16.3	45.1 ± 16.2	42.0 ± 12.6	34.1 ± 8.0	<0.001
Male	929 (31.9%)	1593 (51.1%)	1747 (52.9%)	2284 (65.3%)	<0.001	345 (35.3%)	514 (66.8%)	428 (72.9%)	291 (73.3%)	<0.001
BMI (kg/m^2^)	21.7 ± 2.0	21.3 ± 2.0	20.9 ± 2.0	20.6 ± 1.9	<0.001	22.7 ± 1.6	22.5 ± 1.7	22.2 ± 1.8	22.0 ± 1.9	<0.001
SBP (mmHg)	125.5 ± 17.2	120.1 ± 15.8	116.6 ± 15.1	114.3 ± 14.1	<0.001	127.9 ± 16.1	126.2 ± 15.4	124.2 ± 15.6	122.9 ± 16.2	<0.001
DBP (mmHg)	74.6 ± 10.2	72.5 ± 10.1	70.7 ± 9.7	69.5 ± 9.4	<0.001	77.5 ± 10.1	76.6 ± 10.1	76.3 ± 11.0	75.4 ± 11.0	0.005
ALP (U/L)	73.0 (61.0–86.0)	69.0 (56.0–82.7)	65.6 (53.6–79.0)	63.4 (52.0–78.0)	<0.001	76.0 (63.0–91.0)	75.0 (65.0–88.0)	75.0 (61.0–90.0)	70.0 (59.0–84.4)	<0.001
GGT (U/L)	23.0 (18.0–32.0)	20.0 (15.0–29.4)	19.0 (14.0–29.0)	17.0 (12.0–27.4)	<0.001	31.0 (23.0–48.0)	31.0 (23.0–49.7)	31.0 (21.0–53.0)	29.0 (18.1–49.0)	<0.001
ALT (U/L)	17.0 (13.0–23.0)	16.0 (12.0–23.0)	15.9 (11.0–23.0)	14.5 (10.0–22.6)	<0.001	20.3 (15.0–28.0)	22.0 (16.0–29.0)	21.0 (15.0–28.6)	19.0 (13.0–30.0)	0.002
AST (U/L)	24.1 ± 11.6	22.6 ± 8.2	22.1 ± 8.7	20.9 ± 8.2	<0.001	24.8 ± 8.3	25.2 ± 9.0	24.8 ± 10.0	23.0 ± 7.6	<0.001
ALB (g/L)	44.4 ± 2.9	44.4 ± 2.7	44.3 ± 2.6	44.2 ± 2.6	0.075	44.8 ± 2.6	44.9 ± 2.6	44.7 ± 2.6	44.7 ± 2.7	0.350
GLB (g/L)	29.3 ± 4.2	29.4 ± 3.8	29.6 ± 3.7	29.5 ± 3.6	0.014	29.5 ± 3.8	29.3 ± 3.9	29.6 ± 3.5	29.8 ± 3.8	0.144
TB (μmol/L)	12.9 ± 5.0	12.2 ± 4.8	11.8 ± 5.0	11.6 ± 4.7	<0.001	12.6 ± 5.1	12.5 ± 4.7	12.0 ± 5.1	11.3 ± 4.7	<0.001
DBIL (μmol/L)	2.3 (1.7–3.1)	2.2 (1.5–3.0)	2.2 (1.5–3.0)	2.2 (1.5–3.0)	<0.001	1.9 (1.3–2.7)	2.0 (1.3–2.8)	1.8 (1.2–2.6)	1.9 (1.2–2.6)	0.198
BUN (mmol/L)	5.1 ± 1.4	4.6 ± 1.2	4.4 ± 1.2	4.2 ± 1.1	<0.001	4.9 ± 1.4	4.4 ± 1.1	4.4 ± 1.2	4.2 ± 1.1	<0.001
eGFR (mL/min⋅1.73 m^2^)	69.2 ± 10.3	91.7 ± 4.8	108.2 ± 4.8	127.8 ± 8.3	<0.001	68.9 ± 10.3	91.8 ± 4.8	107.7 ± 4.7	126.6 ± 8.0	<0.001
Scr (μmol/L)	98.0 ± 15.8	80.1 ± 12.1	69.8 ± 10.8	69.8 ± 10.8	<0.001	98.9 ± 15.8	84.4 ± 12.3	74.5 ± 11.5	62.7 ± 10.3	<0.001
FPG (mmol/L)	5.2 ± 0.5	5.1 ± 0.5	5.0 ± 0.4	4.9 ± 0.4	<0.001	5.2 ± 0.5	5.2 ± 0.5	5.2 ± 0.5	5.0 ± 0.5	<0.001
TC (mmol/L)	4.6 ± 0.7	4.6 ± 0.7	4.6 ± 0.7	4.5 ± 0.7	<0.001	4.9 ± 0.7	4.9 ± 0.7	4.9 ± 0.7	4.9 ± 0.7	0.862
TG (mmol/L)	1.1 ± 0.3	1.0 ± 0.3	1.0 ± 0.3	0.9 ± 0.3	<0.001	2.3 ± 0.5	2.3 ± 0.6	2.3 ± 0.5	2.2 ± 0.5	0.008
HDL-c (mmol/L)	1.5 ± 0.3	1.5 ± 0.4	1.6 ± 0.4	1.5 ± 0.4	<0.001	1.2 ± 0.3	1.2 ± 0.3	1.3 ± 0.3	1.3 ± 0.3	<0.001
LDL-c (mmol/L)	2.3 ± 0.5	2.3 ± 0.5	2.2 ± 0.5	2.2 ± 0.4	<0.001	2.4 ± 0.4	2.4 ± 0.4	2.4 ± 0.4	2.4 ± 0.4	0.044

Values are n (%) or mean ± SD or median (quartile).

BMI, body mass index; SBP, systolic blood pressure; DBP, diastolic blood pressure; ALP, alkaline phosphatase; GGT, γ-glutamyl transpeptidase; ALT, alanine aminotransferase; AST, aspartate aminotransferase; ALB, albumin; GLB, globulin; TC, total cholesterol; TG, triglyceride; HDL-C, high-density lipoprotein cholesterol; LDL-C, low-density lipid cholesterol; BUN, serum urea nitrogen; Scr, serum creatinine; FPG, fasting plasma glucose; eGFR, estimated glomerular filtration rate; DBIL, direct bilirubin; TB, total bilirubin.

**FIGURE 2:**
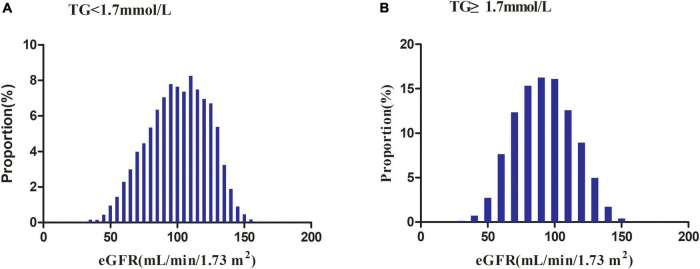
Distribution of eGFR. It presented a normal distribution in the range from 30.19 to 167.11 ml/min per 1.73 m^2^, with an average of 100.69 ml/min per 1.73 m^2^ in the TG < 1.7 mmol/L group. While in the TG ≥ 1.7 mmol/L group, eGFR presented a normal distribution in the range from 31.19 to 162.59 ml/min per 1.73 m^2^, with an average of 92.07 ml/min per 1.73 m^2^.

Figure 2 illustrated the distribution of eGFR levels among two TG groups. A normal distribution was observed during the TG < 1.7 mmol/L group, with an average of 100.69 ml/min per 1.73 m^2^. While in the TG ≥ 1.7 mmol/L group, the eGFR was distributed normally from 31.19 to 162.59 ml/min per 1.73 m^2^, with an average of 92.07 ml/min per 1.73 m^2^.

**FIGURE 3:**
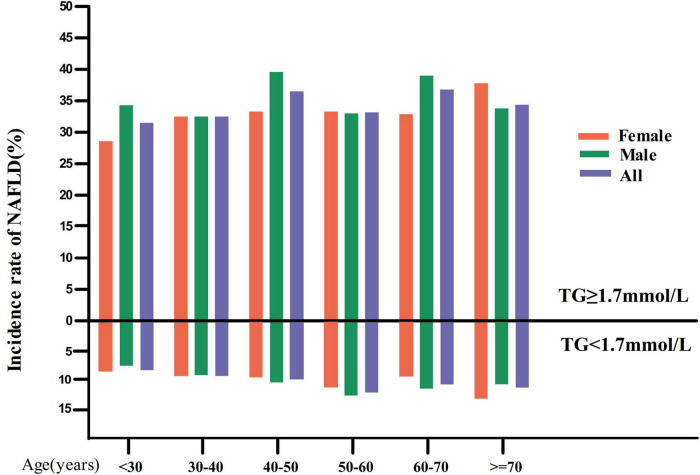
NAFLD incidence rate of age stratification by 10 intervals. Image showed that in the TG < 1.7 mmol/L group, in age stratification by 10 intervals, female subjects had a higher incidence of NAFLD than male subjects within the age range of fewer than 40 years and higher than 70 years old. It also found that males (except for those over 60 years old) and females (except those between 60 and 70) showed an increased incidence of NAFLD with age. While in the TG ≥ 1.7 mmol/L group, in age stratification by 10 intervals, male subjects had a higher incidence of NAFLD than male subjects except in the age range from 30 to 40, 50–60, and over 70 years old.

In the TG < 1.7 mmol/L group, in age stratification by 10 intervals, female subjects had a higher incidence of NAFLD than male subjects within the age range of fewer than 40 years and higher than 70 years old (Figure 3). In addition, males (except for those over 60 years old) and females (except those between 60 and 70) showed an increased incidence of NAFLD with age.

While in the TG ≥ 1.7 mmol/L group, in age stratification by 10 intervals, male subjects had a higher incidence of NAFLD than male subjects except in the age range from 30 to 40, 50–60, and over 70 years old (Figure 3).

#### The incidence rate of non-alcoholic fatty liver disease

Table 2 revealed that during a median follow-up of 35.83 months, 2,077 (13.35%) participants developed NAFLD. There was a cumulative incidence rate of 47.49 per 1,000 person-years for all persons. In particular, in the TG < 1.7 mmol/L group, the cumulative incidence of the four eGFR groups was 55.09, 40.18, 24.12, and 14.72 per 1,000 person-years, respectively. The incidence rate of each eGFR group was 14.67% (13.39–15.96%), 10.94% (9.85–12.04%), 6.94% (6.07–7.80%), and 4.49% (3.80–5.18%), respectively. While in the TG ≥ 1.7 mmol/L group, the cumulative incidence of the four eGFR groups was 151.89, 130.56, 115.79, and 75.44 per 1,000 person-years, respectively. The incidence rate of each eGFR group was 37.93% (34.89–40.98%), 34.98% (31.60–38.36%), 31.86% (28.08–35.64%), and 24.18% (19.95–28.41%), respectively.

**TABLE 2:** Incidence rate of incident NAFLD.

eGFR	Participants (*n*)	NAFLD events (*n*)	Incidence rate (95% CI) (%)	Per 1,000 person-year
Total	15,555	2,077	13.35 (12.82–13.89)	47.49
**TG < 1.7 mmol/L**				
Q1 (<82.88)	2,910	424	14.67 (13.39–15.96)	55.09
Q2 (82.88–99.70)	3,116	341	10.94 (9.85–12.04)	40.18
Q3 (99.70–116.56)	3,302	229	6.94 (6.07–7.80)	24.12
Q4 (≥116.56)	3,496	157	4.49 (3.80–5.18)	14.72
*P* for trend			<0.0001	
**TG ≥ 1.7 mmol/L**				
Q1 (<82.88)	978	371	37.93 (34.89–40.98)	151.89
Q2 (82.88–99.70)	769	269	34.98 (31.60–38.36)	130.56
Q3 (99.70–116.56)	587	187	31.86 (28.08–35.64)	115.79
Q4 (≥116.56)	397	96	24.18 (19.95–28.41)	75.44
*P* for trend			<0.0001	

eGFR, estimated glomerular filtration rate (mL/min⋅1.73 m^2^); NAFLD, non–alcoholic fatty liver disease.

**FIGURE 4:**
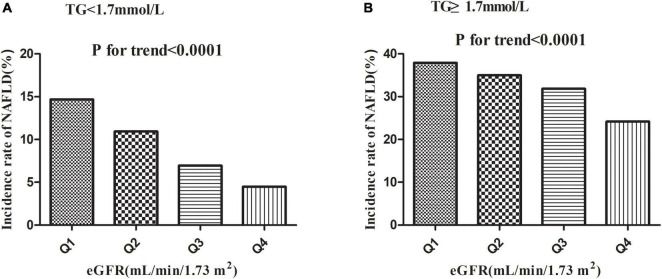
Incidence of NAFLD according to the quartiles of eGFR. Incidence rates of NAFLD were lower among participants with high eGFRs than among those with low eGFRs (*P* < 0.0001 for trend) no matter which group in TG.

Incidence rates of NAFLD were lower among participants with high eGFRs than among those with low eGFRs (*P* < 0.0001 for trend), no matter which group in TG (Figure 4). At the same time, it should be noted that the incidence rate of NAFLD was higher in participants with hypertriglyceridemia than in those without hypertriglyceridemia, regardless of the eGFR grouping.

### Association between triglyceride and estimated glomerular filtration rate

**FIGURE 5:**
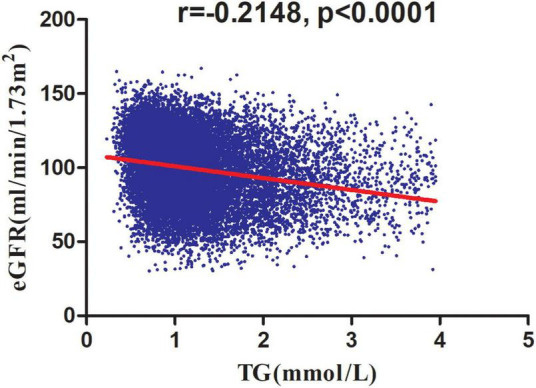
Correlation analysis of TG and eGFR. Correlation analysis results showed that TG was negatively correlated with eGFR (*r* = -0.2148, *p* < 0.0001).

Table 3 showed the association of TG with eGFR in the entire cohort. We used a linear regression model to evaluate the associations between TG and eGFR. In fully adjusted model, TG showed a negative association with eGFR (β = -4.93, 95% confidence interval (CI): -5.45 to -4.41, *P* < 0.0001). We also handled TG as a categorical variable for sensitivity analysis and observed the same trend. Compared with participants without hypertriglyceridemia, people with hypertriglyceridemia had an eGFR decrease of 4.04 ml/min per 1.73 m^2^. Simultaneously, we found that TG was negatively correlated with eGFR by correlation analysis (*r* = -0.2148, *p* < 0.0001) (Figure 5).

**TABLE 3:** Association between TG and eGFR in the entire cohort.

TG (mmol/L)	Crude model (β, 95% CI, *P*)	Model I (β, 95% CI, *P*)	Model II (β, 95% CI, *P*)
TG	-7.99 (-8.56, -7.42) <0.0001	-5.02 (-5.51, -4.52) <0.0001	-4.93 (-5.45, -4.41) <0.0001
**TG group**			
<1.7	Ref.	Ref.	Ref.
≥1.7	-8.62 (-9.55, -7.69) <0.0001	-4.61 (-5.39, -3.82) <0.0001	-4.04 (-4.83, -3.25) <0.0001
**TG group**			
≥1.7	Ref.	Ref.	Ref.
<1.7	8.62 (7.69, 9.55) <0.0001	4.61 (3.82, 5.39) <0.0001	4.04 (3.25, 4.83) <0.0001

Crude model: we did not adjust other covariants. Model I: we adjusted age, sex, BMI, SBP, DBP. Model II: we adjusted age, sex, BMI, SBP, DBP, ALT, ALB, GLB, BUN, FBG, HDL-c, LDL-c.

CI, confidence; Ref, reference; eGFR, estimated glomerular filtration rate (mL/min⋅1.73 m2); TG, triglycerides (mmol/L).

### The association of estimated glomerular filtration rate and triglyceride with incident non-alcoholic fatty liver disease

The Cox proportional-hazards regression model was used to construct three models examining the relationship between eGFR and incident NAFLD (Table 4). The unadjusted model (Crude model*^a^*) showed that an increase in eGFR of 1 mL/min⋅1.73 m^2^ was associated with a 2.1% reduction in the risk of NAFLD (HR = 0.979, 95% CI: 0.977–0.981). As a result of only adjusting for demographic factors in Model I*^a^*, each additional mL/min⋅1.73 m^2^ of eGFR decreased the risk of NAFLD by 1.5% (HR = 0.985, 95% CI: 0.982–0.987). The fully adjusted model (Model II*^a^*) revealed that each additional mL/min⋅1.73 m^2^ of eGFR was associated with a 1.6% decline in NAFLD risk (HR = 0.984, 95% CI: 0.982 to 0.987). To verify the robustness of the results, a sensitivity analysis was addressed. By converting eGFR into a categorical variable (according to quartiles), we re-tested the Cox proportional-hazards regression equation using the categorically changed eGFR. Based on these results, the effect sizes (HRs) between the groups were equidistant. The trend was in agreement with the result when eGFR was considered a continuous variable.

**TABLE 4:** Association between eGFR and TG with risk of incident NAFLD.

eGFR, mL/min⋅1.73 m^2^	Crude model	Model I^a^	Model II^a^
			
	HR (95% CI) *P*-value	HR (95% CI) *P*-value	HR (95% CI) *P*-value
eGFR	0.979 (0.977, 0.981) <0.0001	0.985 (0.982, 0.987) <0.0001	0.984 (0.982, 0.987) <0.0001
**eGFR Quartile**			
<82.88	Ref.	Ref.	Ref.
≥82.88, <99.70	0.709 (0.638, 0.788) <0.0001	0.788 (0.706, 0.879) <0.0001	0.775 (0.694, 0.866) <0.0001
≥99.70, <116.56	0.444 (0.394, 0.500) <0.0001	0.599 (0.528, 0.679) <0.0001	0.603 (0.530, 0.687) <0.0001
≥116.56	0.238 (0.207, 0.275) <0.0001	0.369 (0.315, 0.431) <0.0001	0.382 (0.325, 0.449) <0.0001
*P* for trend	<0.0001	<0.0001	<0.0001

**TG, mmol/L**	**Crude model^b^**	**Model I^b^**	**Model II^b^**

TG	2.552 (2.435, 2.676) <0.0001	1.838 (1.743, 1.937) <0.0001	1.582 (1.490, 1.681) <0.0001
**TG group**			
<1.7	Ref.	Ref.	Ref.
≥1.7	3.952 (3.624, 4.309) <0.0001	2.235 (2.044, 2.445) <0.0001	1.732 (1.574, 1.905) <0.0001
**TG group**			
≥1.7	Ref.	Ref.	Ref.
<1.7	0.253 (0.232, 0.276) <0.0001	0.447 (0.409, 0.489) <0.0001	0.578 (0.525, 0.635) <0.0001

The crude model was adjusted for none.

Adjust I^a^ model was adjusted for: age, sex, BMI, SBP, DBP.

Adjust II^a^ model was adjusted for: age, sex, TG, ALT, ALB, GLB, BUN, GLU, HDL-c, LDL-c, BMI, SBP, DBP.

Adjust I^b^ model was adjusted for: age, sex, BMI, SBP, DBP.

Adjust II^b^ model was adjusted for: age, sex, eGFR, ALT, ALB, GLB, BUN, GLU, HDL-c, LDL-c, BMI, SBP, DBP.

CI, confidence interval; Ref, reference; HR, hazard ratio.

We also constructed three models using the Cox proportional-hazards regression model to explore the relationship between TG and incident NAFLD. In the unadjusted model (Crude model*^b^*), an increase of 1 mmol/L of TG was connected with a 1.55 times increase in the risk of NAFLD (HR = 2.552, 95% CI: 2.435–2.676). In the minimally-adjusted model (Model I*^b^*), when we only adjusted for demographic variables, each additional mmol/L of TG increased by 83.8% in the risk of NAFLD (HR = 1.838, 95% CI: 1.743–1.937). In the fully adjusted model (Model II*^b^*), each additional mmol/L of TG was accompanied by a 58.2% increase in NAFLD (HR = 1.582, 95% CI: 1.490–1.681). We also transformed the TG into a categorical variable (according to the presence or absence of HTG) and then put it back into the Cox proportional-hazards regression equation. After adjusting confounding variables, we found that participants with HTG had a 73.2% increased risk of NAFLD (HR = 1.732, 95% CI: 1.574–1.905). The results suggested that TG is positively associated with NAFLD (Table 4).

**FIGURE 6:**
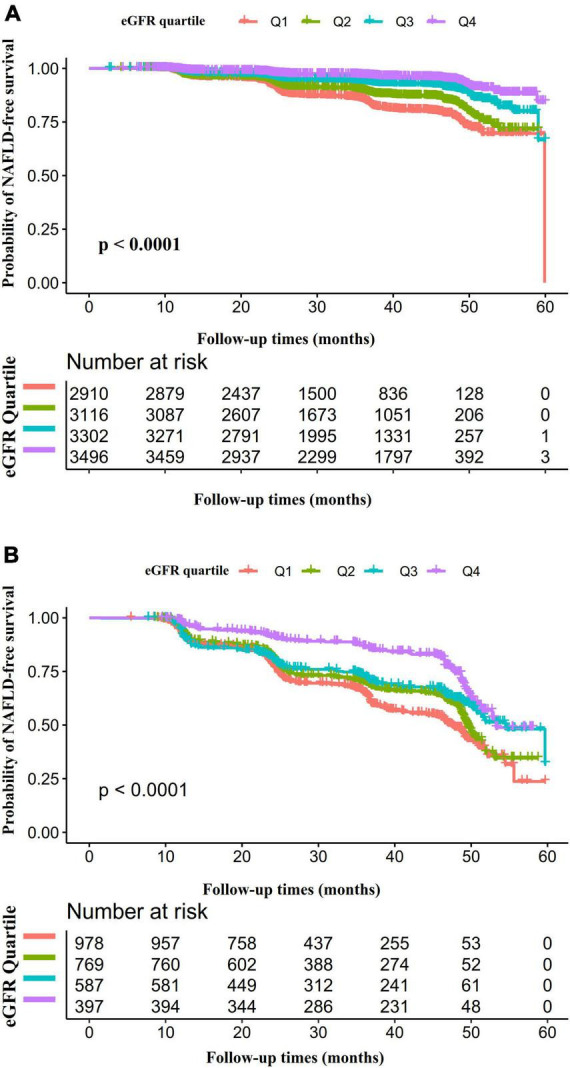
Kaplan–Meier event-free survival curve. A Kaplan–Meier plot depicted NAFLD-free survival probability stratified by the eGFR subgroup in each TG group. The probability of NAFLD-free survival was significantly different among the eGFR subgroups (log-rank test, *p* < 0.0001). NAFLD-free survival probability increased as eGFR increased, suggesting that people with the highest eGFR had the lowest risk of developing NAFLD no matter which group in TG **(A,B)**. We also found that the probability of NAFLD-free survival was higher in the TG < 1.7 mmol/L group than in the HTG group, regardless of the eGFR subgroups.

A Kaplan–Meier plot depicting NAFLD-free survival probability stratified by the eGFR subgroup in each TG group was shown in Figure 6. The probability of NAFLD-free survival was significantly different among the eGFR subgroups (log-rank test, *p* < 0.0001). NAFLD-free survival probability increased as eGFR increased, suggesting that people with the highest eGFR had the lowest risk of developing NAFLD no matter which group in TG (Figures 6A,B). We also found that the probability of NAFLD-free survival was higher in the TG < 1.7 mmol/L group than in the HTG group, regardless of the eGFR subgroups.

### The modification effect of triglyceride on the relationship between estimated glomerular filtration rate and the risk of non-alcoholic fatty liver disease

We performed a stratified univariate and multivariate Cox proportional hazards regression model based on whether participants had hypertriglyceridemia. After adjusting relevant confounders, the results showed a stronger association between eGFR and incident NAFLD in the participants without hypertriglyceridemia (HR = 0.981, 95% CI: 0.978–0.984). In contrast, the weaker association was probed in the population with HTG (HR = 0.986, 95% CI: 0.983–0.989). *P*-value for interaction = 0.0139. When we treated eGFR as a categorical variable (according to quartiles), the results of the stratified Cox proportional hazards model regression analysis were consistent with the results of eGFR as a continuous variable. The above results indicated that TG could modify the relationship between eGFR and NAFLD risk (Table 5).

**TABLE 5:** Effect modification of TG on the association between eGFR and NAFLD risk.

Incident NAFLD	Crude model	Model I	Model II
			
	HR (95% CI) *P*-value	HR (95% CI) *P*-value	HR (95% CI) *P*-value
**eGFR as a continuous variable**			
**TG group**			
<1.7 mmol/L	0.978 (0.976, 0.980) <0.0001	0.984 (0.981, 0.986) <0.0001	0.981 (0.978, 0.984) <0.0001
≥1.7 mmol/L	0.989 (0.986, 0.991) <0.0001	0.989 (0.986, 0.992) <0.0001	0.986 (0.983, 0.989) <0.0001
*P* value for interaction	<0.0001	0.0097	0.0139
**eGFR as a categorical variables**			
**TG group**			
**TG<1.7 mmol/L**			
**eGFR quartile**			
<82.88	Ref.	Ref.	Ref.
≥82.88,<99.70	0.702 (0.609, 0.809) <0.0001	0.808 (0.698, 0.936) 0.0044	0.816 (0.703, 0.946) 0.0071
≥99.70, <116.56	0.407 (0.347, 0.479) <0.0001	0.584 (0.492, 0.693) <0.0001	0.580 (0.487, 0.690) <0.0001
≥116.56	0.235 (0.196, 0.283) <0.0001	0.393 (0.320, 0.482) <0.0001	0.387 (0.313, 0.478) <0.0001
**TG≥1.7 mmol/L**			
**eGFR quartile**			
<82.88	Ref.	Ref.	Ref.
≥82.88, <99.70	0.816 (0.697, 0.955) 0.0112	0.816 (0.691, 0.963) 0.0159	0.731 (0.617, 0.865) 0.0003
≥99.70, <116.56	0.696 (0.583, 0.830) <0.0001	0.726 (0.601, 0.877) 0.0009	0.642 (0.529, 0.779) <0.0001
≥116.56	0.438 (0.350, 0.549) <0.0001	0.447 (0.350, 0.572) <0.0001	0.380 (0.296, 0.488) <0.0001
*P* value for interaction	<0.0001	0.0388	0.0694

Crude model: we did not adjust other covariants. Model I: we adjusted age, sex, BMI, SBP, DBP. Model II: we adjusted age, sex, BMI, SBP, DBP, ALT, ALB, GLB, BUN, FBG, HDL-c, LDL-c.

CI, confidence; Ref, reference; eGFR, estimated glomerular filtration rate (mL/min⋅1.73 m^2^); TG, triglycerides (mmol/L).

Since NAFLD risk was increased in patients with CKD, we excluded participants with eGFR < 60 ml/min/1.73 m^2^ in the sensitivity analysis. 729 (4.69%) participants considered CKD. After adjusting the confounding factors, the results still showed a stronger association between eGFR and incident NAFLD in the participants without hypertriglyceridemia (HR = 0.979, 95% CI: 0.976–0.983). In contrast, the weaker association was probed in the population with TG ≥ 1.7 mmol/L (HR = 0.987, 95% CI: 0.983–0.990). And the *P*-value for interaction = 0.0017. Our sensitivity analyses indicated the robustness of the findings of our study (Table 6).

**TABLE 6:** Effect modification of TG on the association between eGFR and NAFLD risk in participants without eGFR < 60 ml/min/1.73 m^2^ for sensitivity analysis.

Incident NAFLD	Crude model	Model I	Model II
			
	HR (95% CI) *P*-value	HR (95% CI) *P*-value	HR (95% CI) *P*-value
**eGFR as a continuous variable**			
**TG group**			
<1.7 mmol/L	0.974 (0.971, 0.977) <0.0001	0.981 (0.978, 0.984) <0.0001	0.979 (0.976, 0.983) <0.0001
≥1.7 mmol/L	0.987 (0.983, 0.990) <0.0001	0.988 (0.985, 0.992) <0.0001	0.987 (0.983, 0.990) <0.0001
*P* value for interaction	<0.0001	0.0018	0.0017
**eGFR as a categorical variables**			
**TG group**			
**TG < 1.7 mmol/L**			
**eGFR Quartile**			
<82.88	Ref.	Ref.	Ref.
≥82.88, <99.70	0.709 (0.610, 0.824) <0.0001	0.819 (0.703, 0.954) 0.0105	0.837 (0.718, 0.977) 0.0240
≥99.70, <116.56	0.412 (0.348, 0.487) <0.0001	0.594 (0.498, 0.707) <0.0001	0.593 (0.496, 0.708) <0.0001
≥116.56	0.238 (0.197, 0.288) <0.0001	0.397 (0.322, 0.489) <0.0001	0.390 (0.315, 0.484) <0.0001
**TG ≥ 1.7 mmol/L**			
**eGFR Quartile**			
<82.88	Ref.	Ref.	Ref.
≥82.88, <99.70	0.709 (0.610, 0.824) <0.0001	0.834 (0.702, 0.991) 0.0389	0.765 (0.642, 0.911) 0.0027
≥99.70, <116.56	0.702 (0.585, 0.844) 0.0002	0.740 (0.609, 0.899) 0.0025	0.666 (0.546, 0.812) <0.0001
≥116.56	0.443 (0.352, 0.559) <0.0001	0.454 (0.354, 0.583) <0.0001	0.392 (0.303, 0.505) <0.0001
*P* value for interaction	<0.0001	0.0346	0.0647

Crude model: we did not adjust other covariants. Model I: we adjusted age, sex, BMI, SBP, DBP. Model II: we adjusted age, sex, BMI, SBP, DBP, ALT, ALB, GLB, BUN, FBG, HDL-c, LDL-c.

CI, confidence; Ref, reference; eGFR, estimated glomerular filtration rate (mL/min⋅1.73 m^2^); TG, triglycerides (mmol/L).

### The interactive effect of estimated glomerular filtration rate and triglyceride on the risk of non-alcoholic fatty liver disease

**FIGURE 7:**
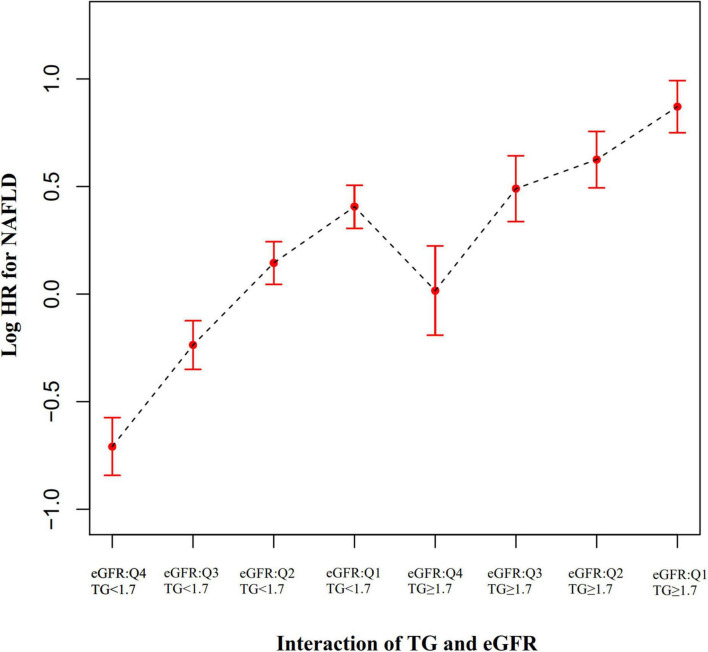
The effect of the interaction of TG and eGFR on the risk of NAFLD. showed the effect of the interaction of TG and eGFR on the risk of NAFLD. We found that the risk of NAFLD in HTG participants with eGFR ≥ 116.56 mL/min⋅1.73 m^2^ (Q4) was lower than that in non-HTG participants with eGFR < 99.70 mL/min⋅1.73 m^2^ (Q2 and Q1).

To explore whether eGFR and TG play an interactive effect on the risk of NAFLD. We divided all participants into eight groups based on eGFR quartiles and hypertriglyceridemia. The eight groups were: non-HTG participants with eGFR ≥ 116.56 ml/min/1.73 m^2^ (Q4), non-HTG participants with eGFR ≥ 99.70 and < 116.56 (Q3), non-HTG participants with eGFR ≥ 82.88 and < 99.70 (Q2), non-HTG participants with eGFR < 82.88 (Q1), HTG participants with eGFR ≥ 116.56 (Q4), HTG participants with eGFR ≥ 99.70 and < 116.56 (Q3), HTG participants with eGFR ≥ 82.88 and < 99.70 (Q2), HTG participants with eGFR < 82.88 (Q1). Using non-HTG participants with eGFR ≥ 116.56 ml/min/1.73 m^2^ (Q4) as a reference, we analyzed the effects of the other seven groups on the risk of NAFLD by univariate and multivariate Cox proportional hazards regression models. The results suggested that the risk of NAFLD was significantly increased in participants with decreased eGFR and hypertriglyceridemia. Specifically, participants with HTG and eGFR < 82.88 ml/min/1.73 m^2^ had the highest risk of developing NAFLD (HR = 4.852 95% CI: 3.943–5.970) (Table 7). It should be noted that the risk of NAFLD in HTG participants with eGFR ≥ 116.56 was lower than that in non-HTG participants with eGFR < 99.70 (Q2 and Q1). The above results suggested that the interaction between eGFR and TG could affect the risk of NAFLD (Figure 7).

**TABLE 7:** Interaction of TG and eGFR and their association with NAFLD.

Exposure	Crude model (HR, 95% CI, *P*)	Model I (HR, 95% CI, *P*)	Model II (HR, 95% CI, *P*)
**Interaction of TG and eGFR**			
Q4 (eGFR ≥ 116.56) and TG < 1.7	Ref.	Ref.	Ref.
Q3 (eGFR ≥ 99.70 < 116.56) and TG < 1.7	1.729 (1.411, 2.119) <0.00001	1.538 (1.253, 1.889) 0.00004	1.602 (1.303, 1.968) <0.00001
Q2 (eGFR ≥ 82.88, <99.70) and TG < 1.7	2.976 (2.463, 3.597) <0.00001	2.196 (1.807, 2.669) <0.00001	2.347 (1.928, 2.857) <0.00001
Q1 (eGFR < 82.88) and TG < 1.7	4.230 (3.521, 5.082) <0.00001	2.780 (2.286, 3.380) <0.00001	3.047 (2.498, 3.718) <0.00001
Q4 (eGFR ≥ 116.56) and TG ≥ 1.7	5.089 (3.947, 6.560) <0.00001	2.523 (1.953, 3.260) <0.00001	2.063 (1.594, 2.672) <0.00001
Q3 (eGFR ≥ 99.70, < 116.56) and TG ≥ 1.7	8.061 (6.519, 9.966) <0.00001	4.015 (3.233, 4.985) <0.00001	3.314 (2.661, 4.127) <0.00001
Q2 (eGFR ≥ 82.88, < 99.70) and TG ≥ 1.7	9.498 (7.799, 11.567) <0.00001	4.452 (3.632, 5.457) <0.00001	3.794 (3.083, 4.669) <0.00001
Q1 (eGFR < 82.88) and TG ≥ 1.7	11.661 (9.671, 14.060) <0.00001	5.332 (4.358, 6.523) <0.00001	4.852 (3.943, 5.970) <0.00001

Crude model: we did not adjust other covariants. Model I: we adjusted age, sex, BMI, SBP, DBP. Model II: we adjusted age, sex, BMI, SBP, DBP, ALT, ALB, GLB, BUN, FBG, HDL-c, LDL-c.

CI, confidence; Ref, reference; eGFR, estimated glomerular filtration rate (mL/min⋅1.73 m^2^); TG, triglycerides (mmol/L).

### The results of subgroup analyses

All prespecified and exploratory subgroups evaluated (Table 8) showed no significant interaction in age, gender, or SBP in the population with TG ≥ 1.7 mmol/L or TG < 1.7 mmol/L. However, interaction effects were detected in variables such as FPG, ALT, DBP, and BMI among participants with TG < 1.7 mmol/L.

**TABLE 8:** Subgroup analyses.

Characteristic	TG < 1.7 mmol/L, HR (95% CI)	*P* for interaction	TG ≥ 1.7 mmol/L, HR (95% CI)	*P* for interacion
Age, years		0.5232		0.5480
<30	0.985 (0.978, 0.991)		0.986 (0.978, 0.994)	
30–40	0.987 (0.982, 0.993)		0.986 (0.979, 0.992)	
40–50	0.981 (0.974, 0.987)		0.986 (0.978, 0.994)	
50–60	0.981 (0.973, 0.989)		0.979 (0.968, 0.989)	
60–70	0.978 (0.966, 0.990)		0.976 (0.964, 0.989	
≥70	0.984 (0.972, 0.996)		0.979 (0.967, 0.992)	
Gender		0.9420		0.2144
Male	0.984 (0.980, 0.988)		0.986 (0.981, 0.991)	
Female	0.984 (0.980, 0.988)		0.982 (0.976, 0.987)	
BMI (kg/m^2^)		0.0132		0.9067
<18.5	0.991 (0.930, 1.057)		0.969 (0.908, 1.033)	
≥18.5, <24	0.977 (0.973, 0.980)		0.982 (0.978, 0.986)	
≥24	0.986 (0.981, 0.992)		0.983 (0.977, 0.988)	
FPG (mmol/L)		0.0170		0.2476
≤6.1	0.981 (0.978, 0.985)		0.984 (0.980, 0.988)	
>6.1	0.995 (0.984, 1.007)		0.977 (0.966, 0.988)	
ALT (U/L)		0.0208		0.4824
≤40	0.984 (0.981, 0.988)		0.983 (0.979, 0.987)	
>40	0.975 (0.967, 0.983)		0.986 (0.978, 0.994)	
SBP (mmHg)		0.2683		0.6130
<140	0.983 (0.980, 0.987)		0.984 (0.980, 0.988)	
≥140	0.987 (0.981, 0.993)		0.982 (0.976, 0.989)	
DBP (mmHg)		0.0336		0.8686
<90	0.982 (0.979, 0.985)		0.984 (0.980, 0.988)	
≥90	0.991 (0.983, 1.000)		0.983 (0.975, 0.992)	

Above model adjusted for age, sex, BMI, SBP, DBP, ALT, ALB, GLB, BUN, FBG, HDL-c, LDL-c.

In each case, the model is not adjusted for the stratification variable.

HR, Hazard ratios; CI: confidence, Ref: reference; eGFR, estimated glomerular filtration rate (mL/min⋅1.73 m^2^); NAFLD, non-alcoholic fatty liver disease.

Specifically, a stronger association between eGFR and NAFLD was observed in DBP < 90 mmHg (HR = 0.982,95% CI:0.979–0.985), BMI ≥ 18.5, < 24 kg/m^2^ (HR = 0.977,95% CI: 0.973–0.980), ALTA > 40 U/L (HR = 0.975,95% CI: 0.967–0.983), and FPG ≤ 6.1 mmol/L (HR = 0.981,95% CI: 0.978–0.985) participants when TG < 1.7 mmol/L. In contrast, the weaker association was probed in those with DBP ≥ 90 mmHg (HR = 0.991,95% CI: 0.983–1.000), FPG > 6.1 mmol/L (HR = 0.995,95% CI: 0.984–1.007), BMI < 18.5 (HR = 0.991,95% CI: 0.930–1.057), or BMI ≥ 24 kg/m^2^ (HR = 0.986,95% CI: 0.981–0.992) in the population with TG < 1.7 mmol/L.

## Discussion

This retrospective cohort study explored the association of eGFR and TG with NAFLD risk. We observed remarkable differences in the association between eGFR and NAFLD risk among subgroups defined by TG. A stronger association between eGFR and incident NAFLD could be found in the participants without hypertriglyceridemia (HR = 0.981, 95% CI: 0.978–0.984, *P* for interaction = 0.0139). In contrast, the weaker association was probed in the population with HTG (HR = 0.986, 95% CI: 0.983–0.989). At the same time, TG and eGFR had an interactive effect in influencing NAFLD risk. In participants with decreased eGFR and hypertriglyceridemia, the risk of NAFLD was significantly increased. Specifically, compared to non-HTG subjects with eGFR ≥ 116.56 ml/min/1.73 m^2^, participants with HTG and eGFR < 82.88 ml/min/1.73 m^2^ had about a fourfold increase in the risk (HR = 3.852 95% CI: 3.943–5.970) of NAFLD.

An American cohort study found that 4.4% of patients with chronic kidney disease developed NAFLD within a median follow-up period of 4.74 years. The researchers also found that CKD3a patients had a higher incidence of NAFLD than those with CKD3b-5. However, our study found that 13.35% of Chinese persons with physical examinations suffered from NAFLD after a median follow-up of 2.986 years. Based on the comparison of the two cohorts, NAFLD was mainly diagnosed in the US population based on elevated ALTs, after excluding viral and alcoholic hepatitis. In contrast, in the present study, NAFLD was diagnosed through ultrasonography. Studies report that 60% of NAFLD patients had normal ALT (52). Perhaps this is the reason for our research’s high incidence of NAFLD. Moreover, due to regional differences in the prevalence of NAFLD, the incidence of NAFLD may differ between China and the United States (53).

Intrahepatic triglyceride content plays an integral role in the pathogenesis of non-obese NAFLD (54). The metabolic syndrome components are closely related to non-obese NAFLD (55). In some studies, non-obese NAFLD has been found to have a genetic predisposition that makes it distinct from obese NAFLD (56). NAFLD in a non-obese population is not uncommon, and genome-wide association studies (GWAS) have identified a single nucleotide polymorphism (rs738409) in the patatin-like phospholipase domain-containing 3 (PNPLA3) gene with the development of NAFLD (57). PNPLA3 encodes a 481 amino acid protein that is expressed in the endoplasmic reticulum and on the surface of lipid droplets in hepatocytes and adipocytes. It has acyl hydrolase activity, which plays a role in the hydrolysis of three major glycerolipids (triacylglycerol, diacylglycerol, and monoacylglycerol), resulting in hepatic triglyceride accumulation (58). Non-obese NAFLD patients have a higher G allele of rs738409 than obese NAFLD patients, and the identified risk allele is strongly associated with increased hepatic fat content, hepatic inflammation, and elevated ALT levels (59). The association between the PNPLA3 G allele and liver fat contact in the non-obese population is significant because it is independent of insulin resistance and other metabolic comorbidities such as obesity and dyslipidemia (60).

**FIGURE 8:**
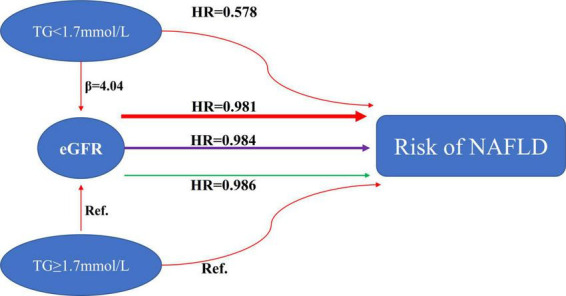
The modification effect of TG on the relationship between eGFR and the risk of NAFLD. We observed remarkable differences in the association between eGFR and NAFLD risk among subgroups defined by TG. A stronger association between eGFR and incident NAFLD could be found in the participants without hypertriglyceridemia (HR = 0.981, 95% CI: 0.978–0.984). In contrast, the weaker association was probed in the population with HTG (HR = 0.986, 95% CI: 0.983–0.989).

According to a recent study, patients with NAFLD and diabetes who have low eGFR are more likely to suffer from liver fibrosis (23). In another study, almost half of the patients with pre-dialysis CKD and non-diabetic CKD on hemodialysis were found to have NAFLD. The study also found an association between greater hepatic steatosis and decreased eGFR and greater CKD stage (48). In addition, the study also found that the degree of NAFLD was significantly positively correlated with serum triglyceride levels (48). Studies have also found that hypertriglyceridemia is independently associated with higher ultrasonographic NAFLD grades (61). A study also found that hypertriglyceridemia was independently associated with a greater prevalence of CKD (62). Our study found that hypertriglyceridemia was inversely associated with eGFR and strongly associated with an increased risk of NAFLD. This was consistent with the results of previous related studies. Furthermore, to the best of our knowledge, the present study analyzed the modification of triglycerides on the relationship between eGFR and NAFLD risk for the first time. When we divided all participants into two groups for subgroup analysis based on whether they had hypertriglyceridemia, a stronger association between eGFR and incident NAFLD could be found in the participants without hypertriglyceridemia (HR = 0.981, 95% CI: 0.978–0.984). In contrast, the weaker association was probed in the population with HTG (HR = 0.986, 95% CI: 0.983–0.989). Combining what was mentioned above and what we describe in Figure 8, people with non-hypertriglyceridemia had a significantly decreased risk of NAFLD and increased eGFR levels compared to non-hypertriglyceridemia. The effect of TG on eGFR and NAFLD is finally manifested in the form of modification through the enhanced degree of association between eGFR and NAFLD risk. The modification effect of TG on the relationship between eGFR and NAFLD has important clinical guiding significance. Clinically, we could prevent NAFLD by delaying the progression of renal function. For patients without hypertriglyceridemia, the effect of delaying the decline of renal function on reducing the risk of NAFLD was more significant. At the same time, our sensitivity analysis found that this effect was not affected by baseline renal functional status.

The mechanism underlying the modification of the relationship between eGFR and NAFLD by triglycerides is still uncertain. The association may, however, be due to insulin resistance. NAFLD and insulin resistance are also interconnected, according to studies (63). Wang et al. found a negative relationship between eGFR and insulin resistance (23). Our previous study also confirmed that eGFR levels were negatively associated with the risk of diabetes in non-CKD populations (64). In addition, hypertriglyceridemia is also associated with increased insulin resistance (65). Therefore, we hypothesized that the triglyceride, eGFR, and NAFLD relationship is linked through insulin resistance.

Furthermore, we also found that the interaction of TG and eGFR can affect the risk of NAFLD. We found that in participants with decreased eGFR and hypertriglyceridemia, the risk of NAFLD was significantly increased. Specifically, participants with HTG and eGFR < 82.88 ml/min/1.73 m^2^ had the highest risk of developing NAFLD (HR = 4.852 95% CI: 3.943–5.970). Since there is an interaction between TG and eGFR in affecting the risk of NAFLD, clinical intervention could be performed from either or both of them to achieve the purpose of reducing the risk of NAFLD. Our findings provide an essential rationale for preventing NAFLD by treatment through delaying renal function decline and lowering triglycerides in the clinic. The interactive effect provides evidence for the first time for the simultaneous management of TG and eGFR in a non-obese Chinese population. The clinical value of this assay is therefore excellent. Findings from this study could be useful for future studies on establishing a risk prediction model for NAFLD. However, it should be noted that the risk of NAFLD in HTG participants with eGFR ≥ 116.56 (Q4) was lower than that in non-HTG participants with eGFR < 99.70 (Q2 and Q1). This result suggests that renal function might effectively inhibit the increased risk of NAFLD caused by hypertriglyceridemia when patients have adequate renal function.

There are some strengths of our study, which are listed below. (1) The sample size was large, which was one of the strengths of our study. (2) We found that TG is an important modifier affecting the relationship between eGFR and incident NAFLD through subgroup analysis and interaction tests. (3) We demonstrated for the first time that the interaction of TG and eGFR could influence the risk of NAFLD. This provided a reference for reducing the risk of NAFLD in clinical practice. (4) Missing data in this study were imputed using multiple imputations. In multiple imputations, we can maximize statistical power and minimize potential bias due to missing covariate information. (5) In this study, we conducted a series of sensitivity analyses to ensure that the results are robust (subgroup analysis, conversion of target-independent variable form, and reanalyzing of the modification effect of TG on the risk relationship between eGFR and NAFLD after excluding participants with eGFR < 60 ml/min/1.73 m^2^). Our results are therefore more reliable.

The following research shortcomings need to be addressed: First, we cannot get an exact causal relationship from this study because it was designed as a cohort observational study. Second, the results can be generalized only to non-obese Chinese with a normal range of LDL-c. In participants with BMI > 25 kg/m^2^, LDL-c > 3.12 mmol/L, or other ethnic populations, the relationship between TG, eGFR, and NAFLD found in this study may not be applicable. In the future, we can consider collecting data from normal weight and obese individuals with normal and abnormal LDL-c levels. This allows us to explore NAFLD and eGFR at various BMI and LDL-c levels. Third, as with all observational studies, even though known confounders such as BMI, SBP, ALT, and FPG were controlled, there may still be unmeasured factors. Socioeconomic, lifestyle behavior (smoking, alcohol use), physical activity, and metabolic disorders (diabetes, hypertension, dyslipidemia, and central obesity) should be adjusted simultaneously in the Cox regression models. In the future, we can design our research to comprehensively collect variables related to socioeconomic, lifestyle behavior, physical activity, and metabolic disorders, so as to analyze the relationship between TG, eGFR and NAFLD more scientifically. In addition, co-morbidities, in particular cardiovascular diseases, are important in NAFLD. We should include them as baseline characteristics in the future. Finally, this study used ultrasonography rather than biopsy to diagnose NAFLD. The main limitation of ultrasound for detecting fatty liver is operator-dependent. The reliability and reproducibility of ultrasonography may not be fully guaranteed. Furthermore, ultrasonography cannot distinguish between steatosis and steatohepatitis. This might affect the proportion of patients with NAFLD diagnosis throughout the study period, subsequently affecting the statistical analysis. However, it is unreasonable to perform routine liver biopsies as a screening or risk assessment test for the general population. Most ultrasound examinations of the health examination population in China are performed by senior and experienced radiologists. The diagnostic performance and observer reliability of ultrasonography were comparable with those of magnetic resonance imaging (MRI) (66). Compared to histology, ultrasound demonstrated 85% sensitivity and 94% specificity in diagnosing moderate-to-severe steatosis (67). It is possible to identify NAFLD early using computed-assisted ultrasonography hepatic/renal ratios and ultrasonography hepatic attenuation rates (68, 69). With a sensitivity of 95% and a specificity of 100%, these values are superior to traditional ultrasound in identifying hepatic steatosis (68, 70). Additionally, this quantitative ultrasound model can be made more reliable and reproducible by standardizing it using a tissue-imitating phantom, albeit further research is required to confirm these results (70). Above all, it is still recommended by current guidelines that ultrasound be used to diagnose NAFLD (71).

## Conclusion

eGFR and TG is independently associated with the risk of NAFLD. The association of eGFR with incident NAFLD is likely to be modified by TG in the Chinese non-obese population. This information extends our existing knowledge to show that eGFR has a much more significant negative effect on NAFLD risk in persons with a normal range of TG. There was also an interaction effect between eGFR and TG in affecting NAFLD risk. In participants with decreased eGFR and HTG, the risk of NAFLD is significantly increased. Our findings provide an essential rationale for preventing NAFLD by treatment through delaying renal function decline and lowering triglycerides in the clinic. The interactive effect provides evidence for the first time for the simultaneous management of TG and eGFR in a non-obese Chinese population.

## Data availability statement

The original contributions presented in this study are included in the article/Supplementary material, further inquiries can be directed to the corresponding authors.

## Ethics statement

The studies involving human participants were reviewed and approved by the Ethics Committee of Wenzhou People’s Hospital. The patients/participants provided their written informed consent to participate in this study. Written informed consent was obtained from the individual(s) for the publication of any potentially identifiable images or data included in this article.

## Author contributions

HH, CC, and YHa developed the manuscript, drafted the research, and conducted the statistical analysis. YHe designed the research and revised the manuscript. All authors approved the final manuscript.

## Footnotes

1
www.datadryad.org

2
http://www.R-project.org

3
http://www.empowerstats.com

## Abbreviations

eGFR: estimated glomerular filtration rate
NAFLD: non-alcoholic fatty liver disease
SBP: systolic blood pressure
FPG: fasting plasma glucose
NASH: non-alcoholic steatohepatitis
Scr: serum creatinine
BMI: body mass index
DBP: diastolic blood pressure
TC: total cholesterol
ALT: alanine aminotransferase
TB: total bilirubin
ALP: alkaline phosphatase
BUN: serum urea nitrogen
TG: triglyceride
ALB: albumin
CKD-EPI: Chronic Kidney Disease Epidemiology Collaboration
AST: aspartate aminotransferase
DBIL: direct bilirubin
LDL-c: low-density lipoprotein cholesterol
GLB: globulin
HDL-c: high-density lipoprotein cholesterol
HR: hazard ratio
GGT: γ -glutamyl transpeptidase
Ref: reference
CI: confidence intervals
WHO: World Health Organization
CKD: chronic kidney disease
GWAS: genome-wide association studies
PNPLA3: patatin-like phospholipase domain-containing 3
MRI: magnetic resonance imaging
HTG: hypertriglyceridemia
IFG: impaired fasting glucose
MAR: missing-at-random
SD: standard deviation
IR: insulin resistance.
